# Profiling of cocaine using ratios of GC-MS peaks

**DOI:** 10.1038/s41598-017-12042-x

**Published:** 2017-09-14

**Authors:** Palle Villesen, Louise Stride Nielsen

**Affiliations:** 1 Bioinformatics Research Centre, Aarhus University, C. F. Møllers Allé 8, 8000 Aarhus C, Denmark; 2 Department of Clinical Medicine, Aarhus University, Palle Juul-Jensens Boulevard 82, 8200 Aarhus N, Denmark; 3 Department of Forensic Medicine, Aarhus University, Palle Juul-Jensens Boulevard 99, 8200 Aarhus N, Denmark

## Abstract

Illicit cocaine seizures are often compared to each other by using gas chromatography-mass spectrometry (GC-MS) data from cocaine alkaloid compounds to determine whether two specimens originate from the same production batch or not. This can provide intelligence or investigative information at the early stages of an investigation or evidence in court. Traditional classification methods assume high stability of all alkaloids, use all of them to calculate the correlation between two profiles and use a threshold to classify samples. Unstable alkaloids will have a strong influence on the performance. We show that comparing each alkaloid target compound individually improves the classification. Unfortunately, it requires normalization and is also sensitive to the stability. Instead we suggest to use ratios of all possible pairwise combinations of the GC-MS peaks. These ratios are scale free and directly comparable between samples. The peaks can be given different weights in the comparison of profiles using appropriate classification methods and we show that randomForest classification using these ratios have a high and reproducible performance in comparison with other methods. The performance of this method is not affected by noise, transformation or normalization and should be considered for future comparison of chromatographic profiles in general.

## Introduction

Statistical comparison of illicit drug gas chromatography-mass spectrometry (GC-MS) profiles is used to determine whether two profiles (specimens) originate from the same production batch^1–8^. Such knowledge can provide intelligence or investigative information at the early stages of an investigation or evidence in court^9^.

In the case of cocaine profiling, eight alkaloid compounds originating from the coca leaves are typically used for statistical comparisons^10, 11^. Unfortunately, some of these alkaloids are unstable over time under certain conditions (e.g. diluted cocaine stored at temperatures above room temperature)^12^. As a consequence, the compound composition in a seizure will slowly change over time and profiles originating from the same production batch will hence begin to look more and more different^12^. Recent work focused on using the residual solvents (trapped in the cocaine crystals) as they appear more stable, but these do not originate from the coca leaf^13^.

Traditionally, comparison of cocaine profiles use scale free comparisons (e.g. Pearson correlation or cosine angle)^1^. If two profiles are correlated above a certain threshold they are considered to be linked. A significant drawback of these methods is that all peaks in the profile are combined into a single variable (the distance). Consequently, this approach makes it impossible to discriminate between stable and unstable peaks.

Instead, we compare each alkaloid compound individually, i.e. define a distance for each of the eight alkaloid peaks and use these alkaloid distances as predictor variables for the classification. It is necessary to normalize each profile so that each peak value is directly comparable between profiles. This is achieved by sample normalization (dividing each peak by the sum of all peaks), where each peak value becomes a fraction of the total. However, if some peaks are highly unstable they will change their value and this will affect the total sum and affect all peaks after normalization. We are not aware of any report using this approach.

Here we also used a different approach for comparing GC-MS profiles by using ratios of all possible pairwise combinations of the GC-MS peaks. The ratios are scale free (as Pearson correlation) and directly comparable between samples. Furthermore, the use of ratios result in multiple variables that can be given different weights in the comparison of profiles using appropriate classification methods. Finally, if a peak is unstable, it will only affect the ratios where this peak is part of the ratio and not all other ratios.

**Figure 1:**
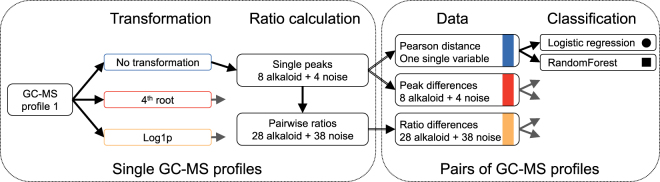
Workflow. Each GC-MS profile was normalized and transformed in three different ways. Both single peaks and all possible pairwise ratios were calculated for each profile. For comparison of two profiles, three different comparisons were performed. This was done for all pairs of profiles (with known linkage status) and submitted to two binary classification methods. All resulting models are a combination of transformation, data and classification method.

In our work presented here (Fig. 1), we used a well described dataset consisting of GC-MS data of eight alkaloids obtained from cocaine samples that were exposed to different environments^12, 13^. To simulate extremely unstable peaks, we added four extra noise peaks to the dataset (each using random peak values) and investigated how these extremely unstable peaks affected the performance of different strategies. We split our data into two sets; training data for training models, estimating performance and model selection and an independent validation dataset only used for validating our results. We analysed the effect of different transformations and different data types (Pearson distance, peak differences and ratio differences). We estimated the performance of two different binary classification methods and showed that randomForest classification using the ratio differences was significantly better than other methods. The performance was not affected by noise, transformation or normalization and should be strongly considered for future comparison of chromatographic profiles in general.

## Results

### The effect of noise on the data

We evaluated the effect of noise on two types of data (peak values or pairwise ratios) and found that the two types of data behaved very differently when noise was added to the data set. For the peak values, the noise affected all peaks after normalization, resulting in very different profiles when visually comparing the profiles with and without noise (Supplementary Fig. 1).

Normalization do not change the ratio between two numbers. Therefore, the ratios of two alkaloid compounds remained unchanged with addition of noise variables (Supplementary Fig. 2). In contrast to the peak values the noise was isolated to ratios with one or two noise variables, resulting a huge variance in these ratios (Supplementary Fig. 2b, right part). Hence, it should be possible to improve classification using the ratios together with appropriate methods that can identify the “pure” ratios and ignore the “noisy” ratios.

### Classification results

**Figure 2:**
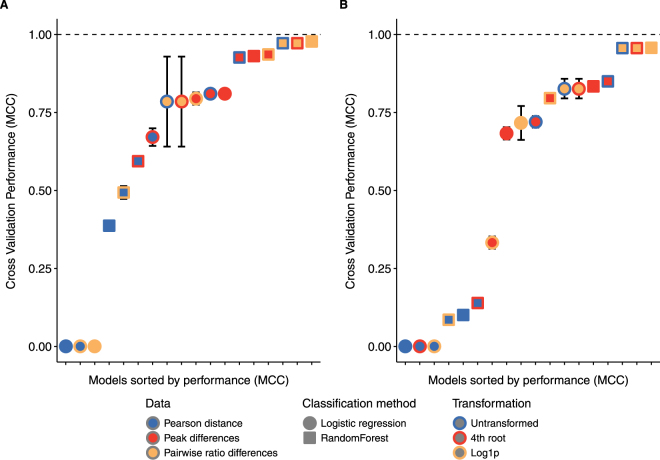
Mean performance +/− SEM of binary classification for all models. The results are shown for increasing mean performance from left to right. Error bars indicate standard error of the mean (SEM). Fill colour indicates data type, shape indicates classification method and outline colour indicates transformation. Performance were measured by Matthews Correlation Coefficient (MCC, y-axis) for the pure alkaloid dataset (**A**) and the data set with added noise (**B**).

All performance results for all models and data are shown in Fig. 2 and Supplementary Table S3. The performance was estimated on the training set using only cross validation or out-of-the-bag estimates. Pearson distance performed the worst regardless of the transformation and classification method. The addition of noise resulted in an even worse performance. Single peak differences showed better performance using logistic regression and was improved further when using randomForest classification but addition of noise to the dataset lowered the performance substantially. Logistic regression on ratio differences either had very low performance or a high variance in the performance. In contrast, randomForest classification on ratio differences performed the best and was not sensitive to noise. Generally, randomForest classification based on single peaks or ratio differences performed very well in the cross validation of the training data. The model with the highest performance both with and without noise used untransformed pairwise ratio differences and randomForest for classification and obtained an estimated MCC very close to 1.0 (but with nearly identical results for all transformations).

### Validation of results

**Figure 3:**
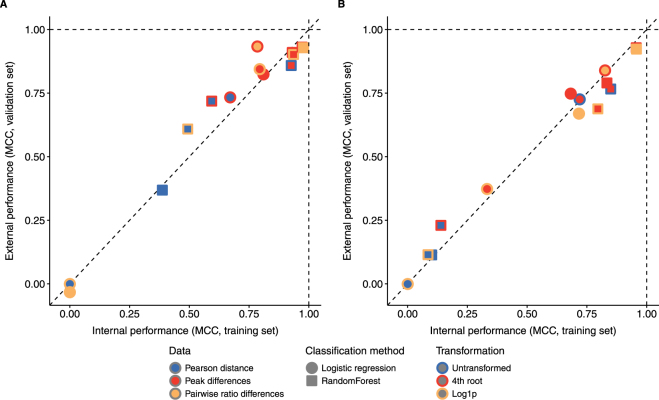
Validation of binary classification. Performance of the different models using different data types (fill colour), different binary classification methods (shape) and different transformations (outline colour). Performance (Matthews Correlation Coefficient) is shown for the training data (x-axis) and validation data (y-axis). The two plots show the pure alkaloid dataset (**A**) and the dataset with the added noise (**B**). Poor models show low internal performance and/or low external performance. Good models will show a high internal and external performance (upper right).

The validation dataset were not involved in any model fitting or model selection but only used for validation. All models were fitted to the training data and the fitted models were then used to predict on the validation dataset (external performance is shown on the y-axis in Fig. 3). Pearson distance models had a low internal performance and/or low external performance (validation), which indicate a lack of predictive power. The high performance of single peak models and models using ratio differences was validated and the single peak models were more sensitive to noise. In general we found a good correlation between internal and external performance.

### Importance of different alkaloids and noise

**Figure 4:**
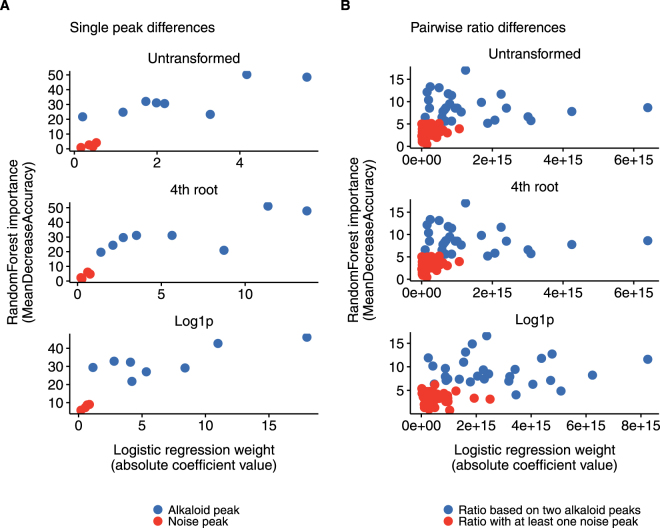
Importance of input variables and the ability to identify noise for the two binary classification methods. The importance assigned to difference variables by logistic regression (x-axis) and randomForest (y-axis). All plots show pure alkaloid variables (blue) and noisy variables (red) for (**A**) single peak differences and (**B**) ratio differences.

For the single peak differences (Fig. 4A), both classification methods gave higher weight to the alkaloid peaks than the noise peaks. For the ratio differences (Fig. 4B), logistic regression (x-axis) had low coefficients for the noise ratios as well as for many of the pure alkaloid ratios (coefficients < 7). RandomForest (Fig. 4B, y-axis) gave high importance to all pure ratios (blue) and low importance to all noisy ratios (with a single exception). In conclusion, randomForest was better at differentiating the real alkaloid variables from the variables filled with random noise.

### Comparison of methods

We used models that return probabilities. This makes it possible to compare different models directly. For logistic regression and Pearson distance, the probability relates directly to the distance (Supplementary Fig. S3). There was a clear overlap between distances for linked and unlinked pairs in the validation data (Supplementary Fig. S3, upper edge) and this overlap was also present in the probabilities (Supplementary Fig. S3, right edge). The overlap of probabilities was decreased when using the ratio differences and randomForest classification (Supplementary Fig. S4). Direct comparison showed that it was different pairs that was difficult to classify correctly for the two strategies. When we analysed the data with added noise there was a clear difference where the noise made classification using Pearson distance nearly impossible, whereas using the ratio differences yielded nearly the same results as without noise (Supplementary Fig. S5).

## Discussion

Many approaches for statistical comparative analysis of illegal drugs in the literature propose normalization as pre-treatment prior to calculation of a distance-based correlation^1, 2, 10^. In this study, a normalization free approach using pairwise ratio differences showed better performance and provided a valid alternative to other methods. Overall, pairwise ratio differences exhibited higher discrimination power compared to either single peak differences or Pearson distance. With the controlled addition of noise to the dataset, the effect was clear (see Fig. 2). External validation showed a high correlation between estimated performance based on cross validation and true performance based on the validation dataset. The better performance of pairwise ratio differences could be a substantial amount of instability in some of the alkaloid variables that affected the Pearson distance as well as the single peak differences (due to normalization). The calculation of all pairwise ratios resulted in some ratios being very pure and optimal for classification (two stable alkaloids) and other ratios being very noisy. The subsequent fitting of a randomForest identified the good predictor variables (the pure ratios) and ignored the noisy ratios resulting in an improved and validated classification performance.

We found that ratio differences and logistic regression had a low and highly variable performance in the training dataset and a low validation performance in the dataset (Fig. 3). This was most likely because logistic regression failed in identifying the true signal (Supplementary Figures S3–S5) and gave high weight to some of the noisy ratios.

In contrast, randomForest gave lower weights to noisy ratios and showed the highest performance and high reproducibility. In conclusion, using randomForest classification on pairwise ratio differences is an applicable approach to obtain a highly discriminative classification of cocaine profile pairs.

Besides better prediction, a normalisation-free approach improves the performance of a profile database as unnecessary calculations are removed and a potential re-calculation of the entire database for each newly added sample is not necessary^2^. The drawback is, however, that a trained model (a fitted random forest) is needed for classification, but distance-based methods also require training data to estimate the threshold to discriminate linked from unlinked.

The presented findings suggest that it is feasible to rely on alkaloids only and obtain reliable results when discriminating between linked and unlinked cocaine seizures even when samples have been stored for months under different conditions. Alkaloids originate directly from the biological source material (the coca leaves) and should be considered the primary information source, whereas the residuals solvents are remnants from the solvents used during the production procedure. Finally, the use of logistic regression or randomForest models provides the investigator with probabilities and a simple likelihood ratio that estimates the certainty of the prediction. We suggest using the ratio differences and randomForest for cocaine comparison purposes as performance seems to be significantly better than distance-based models. In general, the use of ratio differences is highly compatible with comparison of chromatographic profiles and we therefore also recommend this approach for such purposes.

## Methods

### GC-MS profiles

Data used in the present study were also used in two previous studies showing that cocaine GC-MS profiles change over time due to high sensitivity to seizure purity and storage conditions and how residual solvent profiles are useful for improved classification^12, 13^. Five street cocaine seizures were exposed to different experimental conditions and analysed at the beginning of the experiment and subsequently after 6 and 12 months of storage. Furthermore, several groups of specimens stored at room temperature for up to 15 months were also included in the dataset, with each group consisting of linked samples. We extracted some groups and only used these samples for validation of the results. These samples were not included in any model fitting or model selection (Table 1 and Supplementary Table S1). For training data we used the remaining groups and 124 single profiles from different cases and all 124 profiles were presumed to be unrelated to all groups based on the case information. All profiles from the same group were considered to be linked. All pairs with profiles from different groups or one of the 124 single profiles were assumed to be unlinked. Pairs where both profiles came from the 124 single profiles were discarded from the analysis. All groups and the number of samples in each group for both training and validation data can be found in Supplementary Table S1. The number of pairs and the combinations of groups, their training/validation status and linkage status can be found in Supplementary Table S2.

**Table 1:** Number of groups and samples in in the training and validation dataset.

Dataset	Groups	Samples
Training	29	280
Validation	10	68

The cocaine alkaloids were analysed for all samples, and the target compounds including the target and qualifier ions are listed in Table 2. The same 8 cocaine alkaloids were employed in other cocaine classification approaches^1, 10^.

**Table 2:** List of the cocaine alkaloid target compounds and the corresponding target and qualifier ions.

Compound	RRT	Target	Qualifiers
Nonadecane (internal standard)	1.000 (RT 17.300)	57	268, 71, 85
Ecgonine methylester (EME)	0.711	82	83, 96, 271
Ecgonine (EC)	0.793	96	82, 97, 83
Tropacocaine (TROPA)	1.035	124	245, 82, 94
Benzoylecgonine (BENZO)	1.229	240	82, 361, 256
Norcocaine (NOR)	1.243	240	346, 179, 140
Cis-cinnamoylcocaine (CIS)	1.285	82	182, 96, 329
Trans-cinnamoylcocaine (TRANS)	1.376	82	182, 96, 329
3,4,5-Trimethoxycocaine (TMC)	1.630	182	82, 94, 393

### Data analysis

All data analysis was performed in R (version 3.4.0). All scripts are available upon request. Before any subsequent analysis, all profiles were normalized to a total sum of 10^6^ * number of peaks.

### Creation of dataset with noise

We wanted to investigate how the addition of noise would affect the different methods. We simulated four extremely unstable alkaloid peaks by randomly selecting existing peak values from the dataset and adding them as four extra GC-MS peaks resulting in a total of 12 peaks (2/3 real data and 1/3 noise). This approach ensured that these peaks had values of the same distribution as the real GC-MS values, but with no correlation between samples or between peaks.

### Transformation and normalization of data

We evaluated three different transformations (none, 4^th^ root, log1p) followed by final normalization using the row sum (the sum of all peaks). For a given profile, each normalized peak was calculated as in equation (1).

1 $$scale{d}_{i}=\frac{pea{k}_{i}}{pea{k}_{1}\,+\,\ldots \,+\,pea{k}_{8}\,+\,nois{e}_{1}\,+\,\ldots \,+\,nois{e}_{4}}$$

This resulted in 8 single peak variables for the pure alkaloid dataset (8 alkaloids), and 12 single peak variables (8 alkaloids + 4 noise) for the dataset with added noise.

### Pairwise ratios

To create variables that were insensitive to normalization we took all possible pairwise combinations of the peaks and used them to calculate a new variable using equation (2) exemplified by Ecgonine and Tropacocaine.

2 $${{\rm{ratio}}}^{\text{Ecgonine}+{\rm{Tropacocaine}}}=\frac{\text{Ecgonine}\,}{{\rm{Ecgonine}}+{\rm{Tropacocaine}}}$$

The ratio will range from 0 (no Ecgonine, 100% Tropacocaine) to 1 (only Ecgonine). If both peaks are 0 we set the ratio to 0. The total number of pairwise ratios is given by equation (3), where n is the number of peaks.

3 $$number\,of\,ratios=\frac{n\ast (n-1)}{2}$$

For the data presented here, we had 28 ratios (from 8 alkaloids) or 66 ratios from 12 variables (8 alkaloids + 4 noise). In the latter case, 28 ratios were pure alkaloid ratios and the remaining 38 ratios consisted of either one or two noise variables.

### Comparison of profiles

We used three different methods to calculate the difference between two profiles:

#### Pearson distance between two profiles

This is the standard way of comparing cocaine profiles^1^, where the Pearson correlation between the two profiles (r) were converted to a distance by equation (4).

4 $$\text{Pearson}\,\text{distance}\,=\,\frac{1-{r}_{sample1,sample2}}{2}$$

If two profiles are identical, the Pearson correlation will be 1.0, resulting in distance = 0 (range 0–1). We used this single distance variable for classification of the profile pairs.

#### Single peak differences

Here we calculated the difference for each peak (alkaloid) individually using equation (5) exemplified for the Ecgonine peak.

5 $${\rm{Ecgonine}}=\frac{|{{\rm{Ecgonine}}}_{sample1}-{{\rm{Ecgonine}}}_{sample2}|}{{{\rm{Ecgonine}}}_{sample1}+{{\rm{Ecgonine}}}_{sample2}}$$

This resulted in 8 (without noise) or 12 (with noise) peak differences for a pair of profiles (each with 8 or 12 peaks). If two profiles are identical, all the peak differences will be 0. We then used all these peak differences for classification.

#### Pairwise ratios difference

We calculated the difference for each ratio individually using equation (6) exemplified by Ecgonine and Tropacocaine.

6 $${{\rm{rd}}}^{\text{Ecgonine}+{\rm{Tropacocaine}}}=|rati{o}_{sample1}^{\text{Ecgonine}\,+\,{\rm{Tropacocaine}}}-rati{o}_{sample2}^{\text{Ecgonine}\,+\,{\rm{Tropacocaine}}}|$$

The most important difference compared to the peak difference was that the ratio difference is not affected by normalization. As with the other comparison methods, the ratio differences will all be 0 if two profiles are identical. For the data presented here, we had 28 ratio differences (from 8 alkaloids) or 66 ratio differences (from 12 variables, 8 alkaloids + 4 noise) all used for classification. In the latter case, 28 of the 66 ratio differences were based on alkaloid-alkaloid ratios and the remaining 38 consisted of either one or two noise variables and zero or one alkaloids.

### Classification methods

We evaluated two basic binary classification methods to predict the linked or non-linked status of cocaine-seizure pairs: logistic regression and randomForest^13^. Both methods predict the probability that a pair is unlinked (the response variable), and we used the standard threshold of 0.5 to predict whether a pair of profiles were linked or not. The optimal model would give probabilities of 0 (linked) or 1 (unlinked) and make no errors.

### Evaluation of performance

We used the training data to evaluate model performance (internal performance). For logistic regression, we performed 5-fold cross validation to estimate performance. For randomForest, we used the out-of-the-bag estimates of performance that is built into the building of the forest. In this manner, estimates for false positives (FP), false negatives (FN), true positives (TP) and true negatives (TN) (and all derived performance measures) were obtained for both methods. Finally, due to differences in group size (most pairs were unlinked), we used Matthews Correlation Coefficient (MCC) to evaluate performance. The MCC summarizes both true and false positives and negatives and is generally regarded as a balanced measure that can be used even if the classes are of very different sizes. The MCC is a correlation coefficient between the observed and predicted binary classifications. A MCC of 1.0 represents a perfect prediction (FP and FN are both 0), whereas a MCC of 0 is no better than random prediction and −1 indicates total disagreement between prediction and observation (TP and TN are both 0).

7 $$MCC=\frac{(TP\,\ast \,TN)-(FP\,\ast \,FN)}{\sqrt{(TP+FP)\,\ast \,(TP+FN)\,\ast \,(TN+FP)\,\ast \,(TN+FN)}}$$

### Validation of results

We used the training dataset to fit all the different models. The fitted model were then used to predict the linkage status of the validation data without prior knowledge. Performance in the validation set was then obtained by comparison of the prediction to the known linkage status of the validation data (external performance). For the number of pairs and the linked status in the training and validation set, see Table 3.

**Table 3:** The number of profile pairs in the two datasets and their true linkage status.

Dataset	Linkage status	Number of pairs (n)
Training data	linked	422
Training data	unlinked	11668
Validation data	linked	222
Validation data	unlinked	2056

### Measuring importance of different variables

For both peak differences and ratio differences, we used the fitted models to measure how important the different variables were for the model fit. For logistic regression, we used the absolute coefficient values for the fitted models as a measure of importance for both peak differences and ratio differences. For the randomForest models, we used the R function randomForest(…, importance = TRUE) to obtain the mean decrease in classification accuracy for each variable. This analysis was only done for the data with noise included.

### Availability of materials and data

The datasets and scripts are available from the corresponding author on request.

## Electronic supplementary material

Supplementary information

Supplementary tables
