# Supplementary material for: Profiling of cocaine using ratios of GC-MS peaks
Source: Sci Rep. 2017 Sep 14;7:11646. doi: 10.1038/s41598-017-12042-x (PMC5599637; doi:10.1038/s41598-017-12042-x)
Supplement: Supplementary file 1 — Supplementary information [file 41598_2017_12042_MOESM1_ESM.pdf]

Supplementary information

Profiling of cocaine using ratios of GC-MS peaks

Palle Villesen[a,b,*] & Louise Stride Nielsen[a,c]

[a]: Bioinformatics Research Centre, Aarhus University, C. F. Møllers Allé 8, 8000 Aarhus C, Denmark

[b]: Department of Clinical Medicine, Aarhus University

[c]: Department of Forensic Medicine, Aarhus University, Palle Juul-Jensens Boulevard 99, 8200 Aarhus N, Denmark

*: Corresponding author: Palle Villesen, palle@birc.au.dk

Contents

Table S1 – sample sizes 2
Table S2 – pairs of profiles 2
Table S3 – performance results 2
The effect on noise 3
Figure S1 – effect of noise on single peaks 3
Figure S2 – effect of noise on ratios 4
Comparing two methods 4
Figure S3 5
Figure S4 – pure alkaloids 6
Figure S5 – alkaloids with added noise 7

Table S1 – sample sizes

Number of samples in each of the groups of samples and the 124 single profiles. All samples within a group are linked.

The table is attached as excel format in the file Supplementary.Tables.xlsx

Table S2 – pairs of profiles

Pairs of profiles, the number of pairs, their linkage status and training/validation status.

Table S2 is attached as excel format.

The table is attached as excel format in the file Supplementary.Tables.xlsx

Table S3 – performance results

Performance results for all models and data.

Table S3 is attached as excel format.

The table is attached as excel format in the file Supplementary.Tables.xlsx

The effect on noise

Figure S1 – effect of noise on single peaks

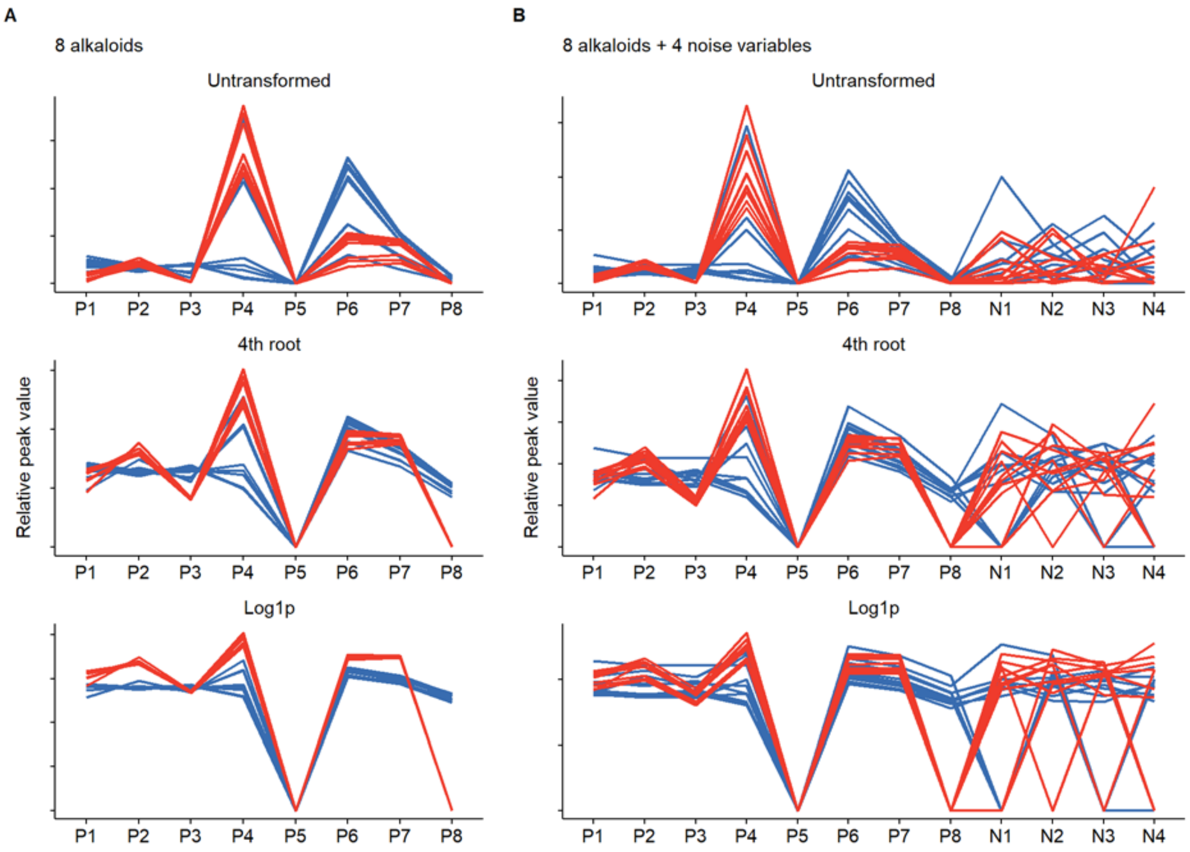

Figure S1. Single peaks and the effect of noise. The figure shows the relative peak values for two groups of linked profiles (red and blue) where all samples within a group are linked. A. Pure alkaloid profiles and different transformations. Some of the peaks can be used to differentiate blue and red samples. B. Addition of random noise variables. All peaks are influenced by the noise when normalizing the profiles and the differentiation of the blue and red samples become more difficult.

Figure S2 – effect of noise on ratios

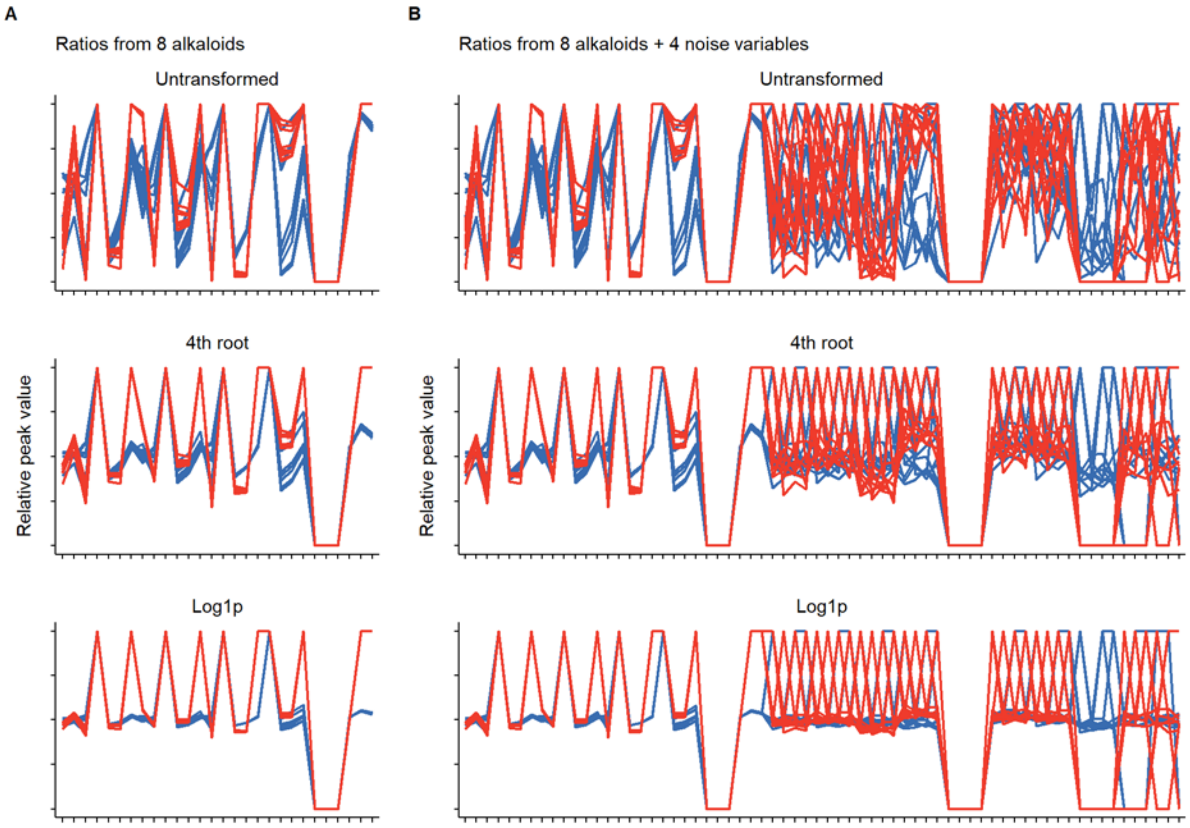

Figure S2. Pairwise ratios and the effect of noise. The figure shows the pairwise ratios for two groups of samples (red and blue) where all samples within a group are linked. A. Pure alkaloid ratios and different transformations. Some of the ratios can easily be used to differentiate blue and red samples. B. Addition of random noise variables. The pure alkaloid ratios remain unchanged (P1-P8) and some can be used to differentiate blue and red samples. The ratios with one or two noise variables (N1-N4) have a high variance and are completely useless for differentiation.

Comparing two methods

Logistic regression on a single variable minimizes the overlap between the two groups. The return value is a probability and normally a threshold of 0.5 is used for classification into one of the two groups. The relationship between distance and probability is shown in Supplementary Figure S3. The comparison with a randomForest model is shown in Supplementary Figures S4 and S5.

Figure S3

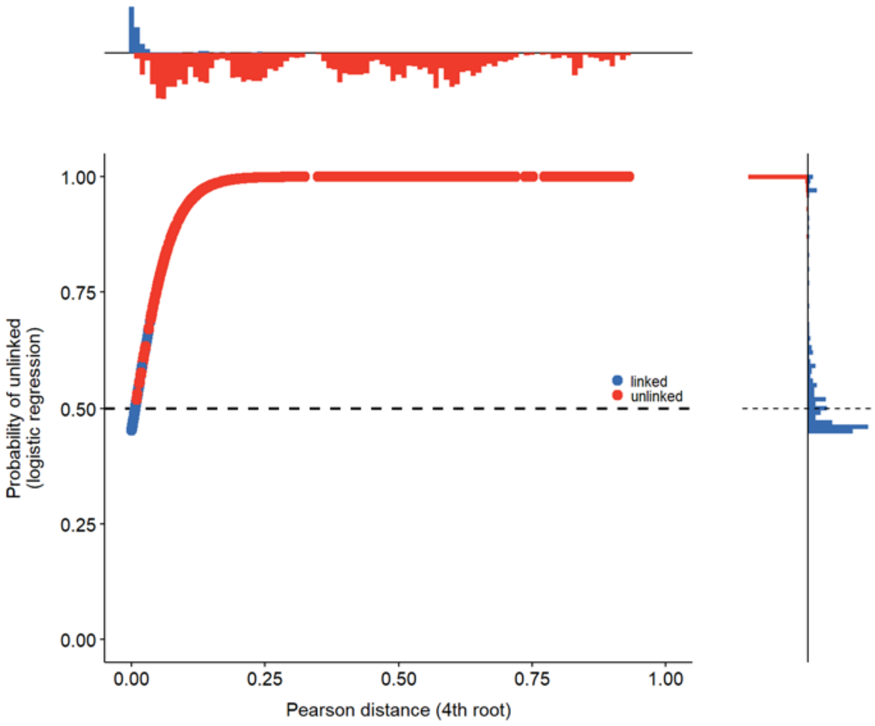

Figure S3. The relationship between Pearson distance (x-axis) and the probabilities returned by logistic regression (y-axis) on the validation data. The distributions are shown at the edges of the plot. The logistic regression model was fitted to the training data and then used to predict on the validation data.

Figure S4 – pure alkaloids

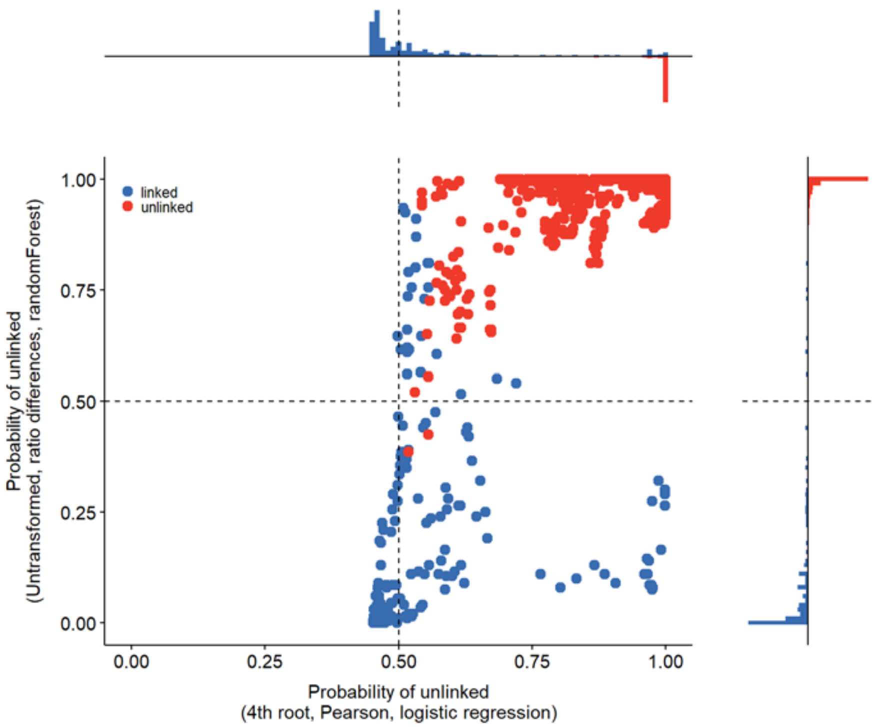

Figure S4. Scatterplot of probabilities from logistic regression on the Pearson distances (x-axis) and the probabilities from RandomForest classification on ratio differences (y-axis). Unlinked pairs (red) in the lower left quadrant are classification errors where both methods fail. Some linked pairs (blue) are misclassified by one method but correctly classified by the other (upper left and lower right quadrant). Distribution of the logistic regression probabilities are shown along the upper edge. Distribution of probabilities returned by RandomForest classification is shown along the right edge of the plot.

Figure S5 – alkaloids with added noise

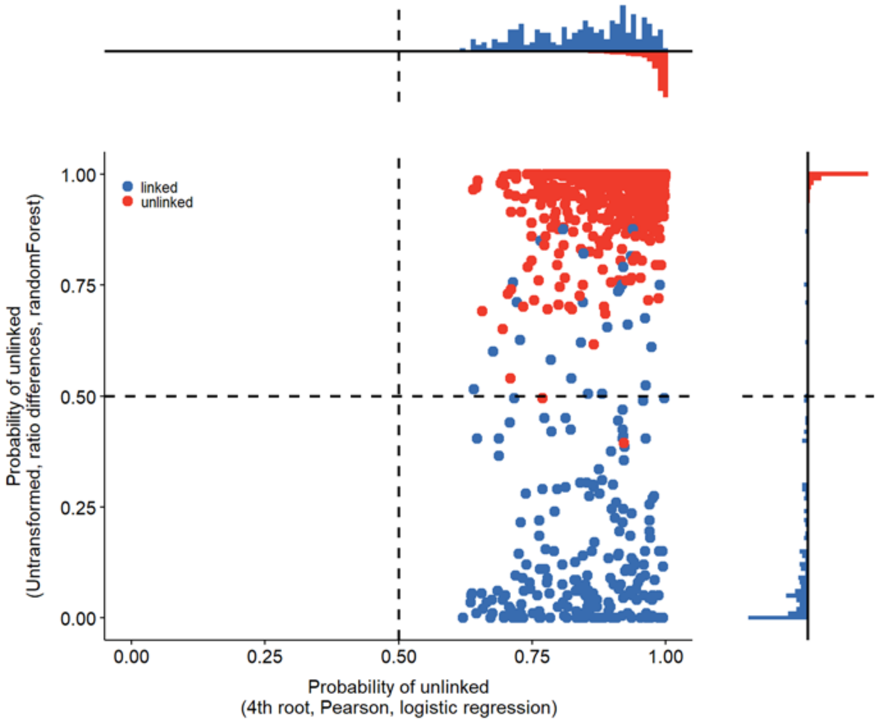

Figure S5. Same description as Figure S4 but for the validation dataset with added noise.
